# Correction

**DOI:** 10.1080/24740527.2026.2616206

**Published:** 2026-02-04

**Article title:** Book of Abstracts

**Journal:** *Canadian Journal of Pain*

**Bibliometrics:** Volume 09, Number 02, 2522029

**DOI:** http://dx.doi.org/10.1080/24740527.2025.2522029

In the 2025 Book of Abstracts, the Hot Topic presentations on pages 2-5 were published with incomplete author attribution.

The online version of the Book of Abstracts has been updated to reflect the complete author attribution for these Hot Topic presentations.

